# Assessment of Machine Learning vs Standard Prediction Rules for Predicting Hospital Readmissions

**DOI:** 10.1001/jamanetworkopen.2019.0348

**Published:** 2019-03-08

**Authors:** Daniel J. Morgan, Bill Bame, Paul Zimand, Patrick Dooley, Kerri A. Thom, Anthony D. Harris, Soren Bentzen, Walt Ettinger, Stacy D. Garrett-Ray, J. Kathleen Tracy, Yuanyuan Liang

**Affiliations:** 1 Department of Population Health, University of Maryland Medical System, Baltimore; 2 Department of Epidemiology and Public Health, University of Maryland School of Medicine, Baltimore; 3 Department of Healthcare Epidemiology, Veterans Affairs Maryland Healthcare System, Baltimore

## Abstract

**Importance:**

Hospital readmissions are associated with patient harm and expense. Ways to prevent hospital readmissions have focused on identifying patients at greatest risk using prediction scores.

**Objective:**

To identify the type of score that best predicts hospital readmissions.

**Design, Setting, and Participants:**

This prognostic study included 14 062 consecutive adult hospital patients with 16 649 discharges from a tertiary care center, suburban community hospital, and urban critical access hospital in Maryland from September 1, 2016, through December 31, 2016. Patients not included as eligible discharges by the Centers for Medicare & Medicaid Services or the Chesapeake Regional Information System for Our Patients were excluded. A machine learning rank score, the Baltimore score (B score) developed using a machine learning technique, for each individual hospital using data from the 2 years before September 1, 2016, was compared with standard readmission risk assessment scores to predict 30-day unplanned readmissions.

**Main Outcomes and Measures:**

The 30-day readmission rate evaluated using various readmission scores: B score, HOSPITAL score, modified LACE score, and Maxim/RightCare score.

**Results:**

Of the 10 732 patients (5605 [52.2%] male; mean [SD] age, 54.56 [22.42] years) deemed to be eligible for the study, 1422 were readmitted. The area under the receiver operating characteristic curve (AUROC) for individual rules was 0.63 (95% CI, 0.61-0.65) for the HOSPITAL score, which was significantly lower than the 0.66 for modified LACE score (95% CI, 0.64-0.68; *P* < .001). The B score machine learning score was significantly better than all other scores; 48 hours after admission, the AUROC of the B score was 0.72 (95% CI, 0.70-0.73), which increased to 0.78 (95% CI, 0.77-0.79) at discharge (all *P* < .001). At the hospital using Maxim/RightCare score, the AUROC was 0.63 (95% CI, 0.59-0.69) for HOSPITAL, 0.64 (95% CI, 0.61-0.68) for Maxim/RightCare, and 0.66 (95% CI, 0.62-0.69) for modified LACE score. The B score was 0.72 (95% CI, 0.69-0.75) 48 hours after admission and 0.81 (95% CI, 0.79-0.84) at discharge. In directly comparing the B score with the sensitivity at cutoff values for modified LACE, HOSPITAL, and Maxim/RightCare scores, the B score was able to identify the same number of readmitted patients while flagging 25.5% to 54.9% fewer patients.

**Conclusions and Relevance:**

Among 3 hospitals in different settings, an automated machine learning score better predicted readmissions than commonly used readmission scores. More efficiently targeting patients at higher risk of readmission may be the first step toward potentially preventing readmissions.

## Key Points

**Question:**

Does a machine learning score better predict hospital readmissions than standard readmission scores?

**Findings:**

In this prognostic study including 14 062 individual patients and 16 649 discharges from 3 hospitals, a machine learning score (the Baltimore score) was better able to predict hospital readmissions compared with standard readmission scores. Area under the receiver operating characteristic curve for the Baltimore score at discharge was 25.5% to 54.9% more efficient compared with commonly used scores with the difference between scores being statistically significant.

**Meaning:**

The findings suggest that an individual hospital-tailored, machine learning–based score better predicts readmissions compared with standard methods.

## Teaser

This prognostic study compares standard readmission risk assessment scores with a machine learning score, the Baltimore score, for predicting 30-day unplanned hospital readmissions calculated in real time.

## Introduction

Hospital readmissions are associated with patient harm and expenses and occur for almost 20% of patients hospitalized in the United States.^1^ Ways to prevent hospital readmissions have focused on identifying patients at greatest risk of being readmitted. Clinicians are poorly able to identify patients who will be readmitted,^2^ and many readmissions are thought to be preventable.^3,4^ Risk factors associated with readmission include polypharmacy use, use of high-risk medications, multiple chronic conditions, and multiple previous admissions.^5^

Commonly used readmission risk-assessment scores rely on a limited set of easily available clinical data that can be used for simple calculations. These include the modified LACE (mLACE) score (calculated at discharge using 4 items: length of stay, acuity of admission, comorbidities, and emergency department visits 6 months before hospital admission), Maxim/RightCare score (calculated 48 hours after admission using a proprietary formula), and HOSPITAL score (calculated at discharge using 7 variables [hemoglobin level, discharge from an oncology service, sodium level, procedure during the index admission, index type of admission, number of admissions during the past 12 months, and length of stay] to categorize patients as low, medium, or high risk).^6^ These scores have rarely been compared directly with each other,^5^ and the discrimination of the scores is moderate.^6^ Some have questioned the effectiveness of the reported better-performing HOSPITAL score.^7^

Modern prediction algorithms have been developed by Amazon, Netflix, and Google. Commonly used approaches rely on using all available data with tools that are complex and multifactorial through machine learning.^8^ Machine learning is often trained around a single customer (eg, Amazon) or a single hospital for health care, thereby best predicting outcomes from that individual or hospital. Unlike traditional modeling, in which a model is derived from 1 cohort and then validated on another cohort, a machine learning algorithm has to be developed to match an individual hospital and then may be validated on future observations within that single hospital. The technique is meant to derive individual rules for each hospital that will change dynamically using a single, generalizable process. With increased adoption of electronic health records (EHRs), machine learning techniques can be applied to health care data. Use of machine learning to predict readmissions has been limited. A non–peer-reviewed publication^9^ at 2 academic centers found readmission prediction at discharge with an area under the receiver operating characteristic curve (AUROC) of 0.75 compared with 0.70 with use of the HOSPITAL score. For specific conditions, such as congestive heart failure, models have identified an AUROC of 0.60 to 0.70 in retrospective cohorts.^10^ Patient composition in these models is significantly associated with the ability to predict readmission.

The objective of our study was to compare the ability of the Baltimore score (B score), an easily implemented machine learning score, for predicting 30-day unplanned readmissions calculated in real time with standard readmission risk-assessment scores in a cohort of patients discharged from 3 hospitals in Maryland.

## Methods

### Study Design and Participants

This prognostic study included consecutive hospital discharges of adult patients from September 1, 2016, through December 31, 2016, at 3 different hospitals in Maryland that are part of the University of Maryland Medical System: University of Maryland Medical Center (a tertiary care academic medical center), Saint Joseph Medical Center (a suburban community medical center), and Maryland Midtown Hospital (an urban safety-net hospital serving mostly indigent patients). Patients not included as eligible discharges by the Centers for Medicare & Medicaid Services (CMS) or the Chesapeake Regional Information System for Our Patients (CRISP) were excluded. Excluded patients were primarily patients who were expected to be readmitted (eg, those receiving scheduled chemotherapy). Because we used the CRISP reporting system to capture readmissions, we could identify all readmissions to hospitals within the Washington, DC; Maryland; and Delaware region. We may have missed a small proportion of readmissions outside the region. This study was approved by the institutional review board at the University of Maryland, Baltimore, which serves as the institutional review board of record for all participating University of Maryland Medical System hospitals, with waiver of informed consent because of the use of retrospective clinical data. The study followed the Strengthening the Reporting of Observational Studies in Epidemiology (STROBE) reporting guideline for cohort studies.^11^

### Predictor Variables

Readmission risk scores were generated based on standard published scales for mLACE and HOSPITAL scores.^6^ The Maxim/RightCare score was collected directly from the hospital administrative data for the one hospital that used it. The experimental B score was based on machine learning algorithms using all available data for each hospital. The model was *bespoke*, meaning that it was trained specifically to fit data from these hospitals. All data were obtained from Epic EHRs (Epic Systems) at each hospital. Initially, more than 8000 possible variables were evaluated against many different model types by using AUROC to determine the best model. The eventual set of key variables used by machine learning is provided in the eAppendix and eTable in the Supplement. The best results were obtained with a weighted combination of gradient-boosting regression trees and convolutional neural network models (chosen via cross-validation) using 382 variables including demographic data. A K-fold cross-validation strategy was used. The model included variables for facility and department to reflect differences in facilities across the hospitals. The model was derived from data collected from September 1, 2014, through August 31, 2016 (before being validated with patients from September 1, 2016, through December 31, 2016). Study periods were chosen based on having complete EHR data and CMS collection of readmissions, and those data were collected before the B score was introduced into clinical practice, being fed back to clinicians for all admissions (January 1, 2017). The score was presented as a rank with a range of 0 to 1. This rank allowed for programmatic hospital planning of resources but did not describe the risk of readmission in the individual patient (rather describing them as in the top 5.0% or 10.0% of risk).

### Validation of Hospital Data

Variables that constitute the HOSPITAL and mLACE scores were validated through medical record review of randomly identified patient records. Among 30 patients and 7 variables, 7 errors were identified for an error rate of 3% (7 of 210 errors). There was no pattern of inconsistent data.

### Study Outcome

The primary study outcome was all-cause hospital readmissions within 30 days of the index visit, excluding planned readmissions, based on CMS and Maryland CRISP definitions for national and statewide reporting.^12,13^ Planned readmissions were not counted as 30-day unplanned readmissions.

### Statistical Analysis

Patient characteristics were summarized using descriptive statistics and were compared between those who had 30-day readmissions and those who did not using the χ^2^ test for categorical variables. For each patient, only the first discharge during the study was used in the primary analysis to ensure that all discharges were independent from each other. For all continuous readmission risk scores of interest, their predictive performances were evaluated using the AUROC and the corresponding 95% CI.^14^ The differences between the AUROCs for various risk scores among the same sample of patients were tested using the nonparametric U statistic method with or without Sidak adjustment for multiple comparisons.^15^ The differences between AUROCs between different groups of patients (ie, by facility or by hospital service [medical vs surgical]) were tested using χ^2^ test statistics. A sensitivity analysis was conducted using all available discharges, including multiple discharges per patient.

On the basis of previously published cutoff values, patients were classified into readmission risk categories. The HOSPITAL score defines low risk as 0 to 4, intermediate risk as 5 to 6, and high risk as 7 or more.^6^ The mLACE score defines low risk as less than 10 and high risk as 10 or more. The Maxim/RightCare score defines low risk as less than 3 and high risk as 3 or more. Sensitivity, specificity, positive predictive value, negative predictive value, and corresponding 95% CIs were computed for all hospitals combined, by facility, and by hospital service.

To compare B score (measured at 48 hours since admission or at discharge) with a given dichotomous risk score and using mLACE score as an example, the following steps were taken. We computed the proportion of patients with mLACE score 10 or more (high risk) among all patients in the study, denoted as *p_1_*. Thus, *p_1_* represented the proportion of patients flagged as high risk based on mLACE score. We computed the proportion of patients with mLACE score 10 or more among those who had 30-day readmissions (sensitivity of dichotomous mLACE score), denoted as *S_1._* We chose a cutoff value for B score, denoted as *C*, such that the proportion of patients with B score greater than *C* among those who had 30-day readmissions equaled *S_1_* (fixed sensitivity). We computed the proportion of patients with B score greater than *C* among all patients (ie, proportion of patients flagged based on B score greater than *C*), denoted as *p_2_*. Improved efficiency was computed as (*p_1_ - p_2_)/p_1_*, and *p_1_* and *p_2_* were compared using the McNemar test.

To further explore the utility of B score, we computed diagnostic statistics (eg, sensitivity, specificity, and positive predictive value) when patients scoring in the top 5.0% (or top 10.0%) by the B score were flagged as high risk. All analyses were completed in Stata/SE, version 15 (StataCorp), and all plots were produced in R, version 3.4.3 (R Core Team).

## Results

From September 1, 2016, to December 31, 2016, a total of 14 062 individual patients were discharged for a total of 16 649 discharges (including repeat admissions) from the 3 participating hospitals. Of these, there were 10 732 unique patients with CMS-eligible discharges for potential unplanned readmissions. Of the 10 732 patients (5605 [52.2%] male; mean [SD] age, 54.56 [22.42] years), 6214 were from hospital 1, 3440 from hospital 2, and 1078 from hospital 3. Unplanned readmissions were more common at hospitals 1 and 3, among African American patients, and at medical hospital services (Table 1).

**Table 1.** Demographics of All 10 732 Patients in the Study Population Comparing Those Without Readmission With Those With Readmission

Characteristic	No. (%)^a^		*P* Value^b^
	Without Readmission (n = 9310)	With Readmission (n = 1422)	
Facility, No. (%)			
Hospital 1, tertiary care (n = 6214)	5337 (85.9)	877 (14.1)	<.001
Hospital 2, community suburban (n = 3440)	3083 (89.6)	357 (10.4)	
Hospital 3, urban (n = 1078)	890 (82.6)	188 (17.4)	
Sex, No. (%)			
Male (n = 5605)	4841 (86.4)	764 (13.6)	.22
Female (n = 5127)	4469 (87.2)	658 (12.8)	
Race/ethnicity, No. (%)			
Black (n = 4004)	3408 (85.1)	596 (14.9)	.001
Hispanic (n = 320)	281 (87.8)	39 (12.2)	
White (n = 6120)	5364 (87.6)	756 (12.4)	
Other or not reported (n = 288)	257 (89.2)	31 (10.8)	
Hospital service, No. (%)			
Medicine (n = 5830)	4970 (85.2)	860 (14.8)	.001
Surgery (n = 4902)	4340 (88.5)	562 (11.5)	

^a^ Percentages are row percentages.

^b^ Comparing the differences between the 2 groups using χ^2^ test.

**Figure 1.**
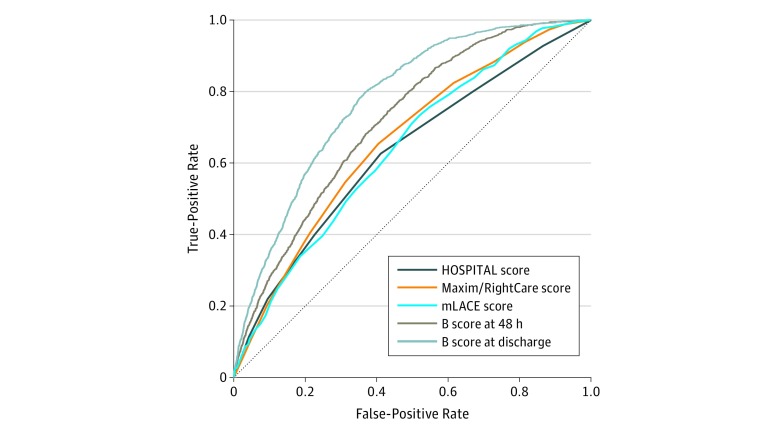
Performance of All Scores Across Continuous Scoring for All Hospitals and First Discharges The modified LACE (mLACE) score was calculated at discharge using 4 items: length of stay, acuity of admission, comorbidities, and emergency department visits 6 months before hospital admission. The Maxim/RightCare score was calculated 48 hours after admission using a proprietary formula. The HOSPITAL score was calculated at discharge using 7 variables (hemoglobin level, discharge from an oncology service, sodium level, procedure during the index admission, index type of admission, number of admissions during the past 12 months, and length of stay) to categorize patients as low, medium, or high risk. B score indicates Baltimore score.

Accuracy of hospital readmission rules varied. Performance of all scores across continuous scoring is shown in Figure 1. Among the 10 732 patients from all hospitals, the AUROC was 0.63 (95% CI, 0.61-0.65) for HOSPITAL score, which was significantly lower than 0.66 for mLACE score (95% CI, 0.64-0.68) (*P* < .001). The B score was significantly better than other scores and was improved significantly when calculated at time points with more data available. Specifically, 48 hours after admission, the AUROC of the B score was 0.72 (95% CI, 0.70-0.73) and increased to 0.78 (95% CI, 0.77-0.79) at discharge (*P* < .001 for both).

Among the 2291 patients from hospital 2 who had a Maxim/RightCare score, the AUROC was 0.63 (95% CI, 0.59-0.69) for HOSPITAL score, 0.64 (95% CI, 0.61-0.68) for Maxim/RightCare score, and 0.66 (95% CI, 0.62-0.69) for mLACE score. Scores were not significantly different from each other; however, the readmission prediction ability was significantly improved using the B score. The AUROC for B score 48 hours after admission was 0.72 (95% CI, 0.69-0.75) and at discharge was 0.81 (95% CI, 0.79-0.84), which was significantly improved compared with the AUROC for mLACE score at discharge (0.66 [95% CI, 0.62-0.69]) (both *P* < .001).

Readmission risk prediction using any score was better in the hospital serving a suburban population (hospital 2) or an academic medical center (hospital 1) than in a critical access hospital (hospital 3). For example, for B score at discharge, the AUROCs were 0.81 (95% CI, 0.79-0.83) for hospital 2, 0.78 (95% CI, 0.76-0.79) for hospital 1, and 0.66 (95% CI, 0.61-0.70) for hospital 3.

Readmission risk prediction using any score was better among surgical patients than medical patients. For B score at discharge, the AUROC was 0.82 (95% CI, 0.80-0.84) among surgical patients and 0.75 (95% CI, 0.73-0.76) among medical patients (*P* < .001).

**Figure 2.**
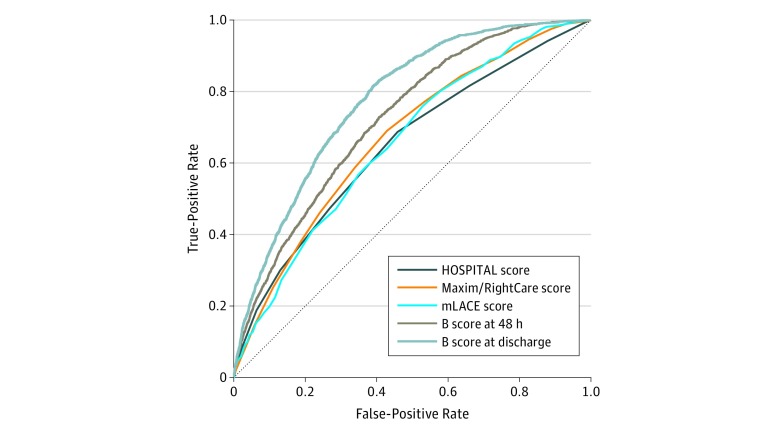
Sensitivity Analysis of All Hospital Discharges, Including Multiple Discharges per Patient Definitions of the scores are given in Figure 1. B score indicates Baltimore score.

The accuracy of hospital readmission rules was similar in a sensitivity analysis of all discharges, including multiple discharges per patient (Figure 2). Among the 12 072 discharges from all 3 hospitals, the AUROC for HOSPITAL score was significantly lower than the AUROC for mLACE score (0.65 [95% CI, 0.64-0.67] vs 0.66 [95% CI, 0.66-0.69]; *P* = .002). The B score was significantly better than other scores. Specifically, the AUROC of the B score was 0.73 (95% CI, 0.71-0.74) 48 hours after admission, which increased to 0.78 (95% CI, 0.77-0.79) at discharge.

Among the 2560 discharges with a nonmissing Maxim/RightCare score, the AUROC was 0.64 (95% CI, 0.61-0.68) for HOSPITAL score, 0.66 (95% CI, 0.63-0.69) for Maxim/RightCare score, and 0.67 (95% CI, 0.64-0.70) for mLACE score. Scores were not significantly different after multiple comparison adjustment; however, the readmission prediction ability was significantly improved using the B score. The AUROC for B score 48 hours after admission was 0.73 (95% CI, 0.71-0.76) and at discharge was 0.81 (95% CI, 0.79-0.83). Compared with mLACE score, the AUROC for B score was significantly improved at both 48 hours after admission and at discharge (both *P* < .001).

Hospital admission rules are often formalized around cutoff scores to aid in their interpretation. When assessed in a dichotomous fashion based on previously published cutoff values (HOSPITAL score, mLACE score, and Maxim/RightCare score), the number of patients flagged, the proportion of readmissions detected (sensitivity), and efficiency between scores are presented in Table 2. When directly compared and by setting the B score to the sensitivity at cutoff values for mLACE score, HOSPITAL score, and Maxim/RightCare score, the B score was better able to identify patients at risk of readmission than other scores (Table 2). In direct comparison with mLACE high vs low risk, we found that mLACE scores identified 54.7% of readmissions by flagging 34.4% of patients. The B score at discharge was able to identify 54.7% of readmissions by flagging 23.6% of patients (31.4% improved efficiency; *P* < .001). In comparison with the HOSPITAL medium/high vs low risk, the B score at discharge was 54.9% more efficient (*P* < .001). In comparison with the Maxim/RightCare score (n = 2291), the B score at 48 hours after admission was 25.5% more efficient (*P* < .001) and the B score at discharge was 50.4% more efficient (*P* < .001).

**Table 2.** Performance of Readmission Scores Using Cutoff Values Among All Patients Across 3 Hospitals^a^

Score	Patients Flagged, %	Readmissions Detected, %^b^	Improved Efficiency, %^c^	*P* Value^d^
mLACE, high vs low	34.4	54.7	31.4	<.001
B score at discharge to detect 54.7% readmitted^e^	23.6			
HOSPITAL, medium/high vs low	25.1	40.2	54.9	<.001
B score at discharge to detect 40.2% readmitted^e^	16.2			
HOSPITAL, high vs medium/low	5.0	11.2	32.0	<.001
B score at discharge to detect 11.2% readmitted^e^	3.4			
Maxim/RightCare^f^	41.5	57.7	25.5	<.001
B score 48 h after admission to detect 57.7% readmitted	30.9			
B score at discharge to detect 57.7% readmitted	20.6		50.4	<.001
B score ≥0.167 at discharge, top 10%	10.0	28.6	37.5^g^	NA
B score ≥0.217 at discharge, top 5%	5.0	16.4	43.1^g^	NA

Abbreviations: B score, Baltimore score; NA, not applicable.

^a^ The modified LACE (mLACE) score was calculated at discharge using 4 items: length of stay, acuity of admission, comorbidities, and emergency department visits 6 months before hospital admission. The Maxim/RightCare score was calculated 48 hours after admission using a proprietary formula. The HOSPITAL score was calculated at discharge using 7 variables (hemoglobin level, discharge from an oncology service, sodium level, procedure during the index admission, index type of admission, number of admissions during the past 12 months, and length of stay) to categorize patients as low, medium, or high risk.

^b^ Sensitivity for identifying readmissions.

^c^ Improved efficiency was computed as (*p_1_–p_2_)/p_1_*, where *p_1_* represents the proportion of patients flagged as high risk based on mLACE; *p_2_* indicates the proportion of patients with B score greater than or equal to the cutoff value among all patients.

^d^ Comparing the percentage of patients flagged at a given sensitivity using the McNemar test.

^e^ The B score being a rank classifier identifies patients by relative risk. Therefore, cutoffs were identified to match the proportion of patients identified by standard scores (sensitivity) or patients identified in the top 5% or 10% of risk. The B score was matched to the percentage of readmissions predicted by standard scores at standard cutoff values for high vs low risk (mLACE, HOSPITAL, and Maxim/RightCare scores).

^f^ Collected at hospital 2 only and measured at 48 hours after admission (n = 2291).

^g^ Indicates percentage readmitted.

These findings suggest that the B score can be used to predict patients at highest risk of readmission. Patients scoring in the top 10.0% of risk by B score at discharge (B score, ≥0.167) had a 37.5% chance of 30-day unplanned readmissions (positive predictive value of 37.5%, sensitivity of 28.3%, and specificity of 92.8%). Likewise, patients scoring in the top 5.0% on B score at discharge (B score, ≥0.217) had a 43.1% chance of readmissions (positive predictive value of 43.1%, sensitivity of 16.2%, and specificity of 96.7%).

## Discussion

Across 3 hospitals in different settings, we found that an automated machine learning approach had better discrimination than commonly used readmission scores. The B score was 25.5% to 54.9% more efficient than comparison scores, meaning that a similar number of readmissions could be prevented by intervening on 25.5% to 54.9% fewer patients and thus better targeting resources. Individual, hospital-specific, machine learning scores derived in a 24-month period were able to best predict readmissions in the 4-month study period.

Predicting readmissions is difficult. However, a machine learning score at discharge had better discriminative ability than other currently used scores in our study. The Maxim/RightCare, mLACE, and HOSPITAL scores performed in a fashion similar to one another, which was not surprising given that they each used a limited number of variables for a simple, nonstochastic prediction of readmissions. Machine learning was adapted for each hospital by using a technique that builds specific rules for each hospital compared with rules such as those in mLACE.^8^ The comparison of readmission scores occurred in the same population as development for the machine learning B score (for each hospital). This factor was an advantage for the B score compared with other scores, which in part explained its better performance; however, the benefit of machine learning is that it is trained for each hospital and weighted for individual characteristics (similar to shaping Netflix recommendations for each individual user). The machine learning derivation of a readmission score occurred for each hospital cohort before validation of the score; therefore, the model was not overfitted. Rather than validating any specific score, we evaluated the technique of machine learning applied to each hospital. Use of machine learning to predict readmissions has been used by Google and reported in the non–peer-reviewed literature to achieve a similar predictive ability (ie, AUROC) to the B score.^9^ Machine learning for identifying readmissions benefits from access to many variables in EHRs and an outcome of readmission to teach the machine learning model. Therefore, associations and predictions can be estimated with more complex processes than past simple scores. With increasing availability of machine learning tools for EHR, prediction rules such as the B score will soon become available in Epic and other EHRs. Multivariable regression modeling with larger data sets has found similar results in terms of predicting readmissions.^16,17^ Calibration analysis was not possible given that the B score is not a probability classifier but a rank classifier that was designed to determine which patients are at the highest risk of readmission to target hospital resources at the administrative level (eg, flagging the top 5.0% or 10.0% of patients with the highest B scores for special risk management or interventions). Future work could improve the B score by providing the probability to describe the actual percentage chance of readmission for an individual patient. Providing actual percentage chance of readmission would be more understandable to clinicians and allow calibration.

Predicting readmission is only one step toward preventing readmissions. Although we used the CMS definition of potentially preventable readmission, the literature would suggest that most of these readmissions are not preventable.^18^ Interventions to prevent readmissions are often labor intensive and costly, including discharge clinics, transitional care, and telemonitoring.^5^ More accurate readmission scores allow for better targeting use of these interventions. The B score was able to identify the same number of readmissions while flagging 25.5% to 54.9% fewer patients for intensive case management.

In our study, prediction was worse with all scores at the critical access hospital and in populations with poor social determinants of health, as well as being worse among medical compared with surgical patients; these findings have been previously reported by other authors.^1,5^ This result has implications for the fairness of CMS penalties for readmission rates and risk adjustment of high-risk populations. Although the B score had access to some social determinants of health such as home zip code, insurance type, and homelessness, the validity of such data in the EHR is uncertain. Further work on predicting and targeting readmission prevention efforts needs to account for social determinants of health.

The B score has become automated at 3 hospitals using Epic EHR in this study and is updated hourly for all patients from admission to discharge. Part of this automation includes monthly model updating as well as yearly feature reevaluation and model retraining to reflect constant changes in health care practice patterns, EHR sophistication, and population demographics. Efforts are now underway to use this prediction score to better target patients at high risk for readmission for interventions. The best presentation of a machine learning approach, the ability to identify preventable readmissions, and ultimately methods to prevent readmission are unknown.

### Limitations

Our study is limited by being performed in 1 US state over a limited time. Furthermore, the predictive ability of the score varied by hospital. Although machine learning was validated at the same hospital where it was developed, this comparison mimics real-life use of machine learning readmission scores that would be developed for each hospital. This score was optimized based on a measure of rank or discrimination (ie, AUROC) rather than a measure of loss or error in terms of the value of the estimated risk. As a result, the B score is an ordinal measure that can sort patients as accurately as possible by risk of readmission, but it does not include a definite probability that would be more useful for individual clinicians (eg, a patient has a 20% risk of readmission).

## Conclusions

We found that a machine learning score, the B score, built from variables in the EHR, had a better discriminative ability to predict readmissions than commonly used readmission scores. This improved prediction may allow for more efficient and targeted use of resources aimed at preventing readmissions.

## Notes

Supplement. **eAppendix**. Baltimore Score Machine Learning Model Development **eTable.** Categories of Features in the Final Machine Learning Model
