# Supplementary material for: Assessment of Machine Learning vs Standard Prediction Rules for Predicting Hospital Readmissions
Source: JAMA Netw Open. 2019 Mar 8;2(3):e190348. doi: 10.1001/jamanetworkopen.2019.0348 (PMC6484642; doi:10.1001/jamanetworkopen.2019.0348)
Supplement: Supplement. — eAppendix. Baltimore Score Machine Learning Model Development eTable. Categories of Features in the Final Machine Learning Model [file jamanetwopen-2-e190348-s001.pdf]

Supplementary Online Content

Morgan DJ, Bame B, Zimand P, et al. Assessment of machine learning vs standard prediction rules for predicting hospital readmissions. *JAMA Netw Open*. 2019;2(3):e190348. doi:10.1001/jamanetworkopen.2019.0348

eAppendix. Baltimore Score Machine Learning Model Development

eTable. Categories of Features in the Final Machine Learning Model

This supplementary material has been provided by the authors to give readers additional information about their work.

© 2019 Morgan DJ et al. *JAMA Network Open.*

eAppendix. Baltimore Score Machine Learning Model Development

The same process was followed over three hospitals using each hospital's individual databases from data collected from September 1, 2014 through August 31, 2016.

The B-score, presented as a rank with a range of 0-1, was produced by a predictive model trained using machine learning techniques and algorithms. The outcome to which this model was trained was all-cause readmissions within 30 days of the index visit, excluding planned readmissions, based on CMS definitions. The model was "bespoke", meaning it was trained specifically to fit data from these hospitals in order to produce more accurate predictions for the relevant patient and provider populations. Even though this model is not, and was not meant to be, a general-purpose tool, the same techniques used to create this model could be used to produce a custom model for any hospital where similar data is available.

To create this predictive model, a wide range of data was extracted from each of the three hospital's Epic-based electronic health record. This data was then cleaned, mapped, and "engineered" to produce features suited to machine learning tasks. Initially almost 9,000 features were produced. Many different model types were trained, optimized, and evaluated using this set of features. During this process the feature set itself was culled using model-specific methods. Each model and subset of features was evaluated using AUROC across several data sets using a K-fold cross-validation strategy to minimize over-fitting and maximize generality.

© 2019 Morgan DJ et al. *JAMA Network Open.*

Best results were obtained with a weighted combination of 500+ Gradient Boosted Regression Trees (GBRT) and a Convolutional Neural Network (CNN), with the final feature set culled to 382 features. The final set of features included several representing facility and department to reflect differences between hospitals.

© 2019 Morgan DJ et al. *JAMA Network Open.*

eTable. Categories of Features in the Final Machine Learning Model

**Feature Category**	**Description**
Admission Type	Inpatient, Outpatient, Emergency, Elective, Direct, etc.
Admitting Diagnosis	ICD Code
Admitting Service	Hospital Service at admission (post-ER)
Admitting Source	Home, Physician Referral, ER, Skilled Nursing Facility, Assisted Living, etc.
Affiliated with a Specific Church	Yes/No
APR DRG Mortality Code	Numeric: 1-5 for this visit, and delta from prior visit max
APR DRG Severity Code	Numeric: 1-5 for this visit, and delta from prior visit max
Basic Measures	Age (at admission), gender, height, weight, and weight change over time
BMI, BSA, and %IBW	Calculated from height and weight, and change from prior visits
Blood	Number of units and types of units infused
Breathing Assistance	Yes/No and Type, this visit and prior visit: Nasal cannula, Face mask, Tracheostomy, BiPap (not ventilator)
Central Line	Categorized by line type, this visit and prior visits
Characterization of Home Address	Several variables extracted from 2010 Census and subsequent ACS data (U.S. Census Bureau)
Charlson Comorbidity Index	Charlson Comorbidity Index
Diagnostic History	ICD Codes from prior visits
Discharge Disposition	Home, Skilled Nursing Facility, Assisted Living, Acute Care Facility, Hospice, AMA, etc.
Discharge Service	Hospital Service at discharge
Discharge Status	Alone, Accompanied, Ambulance, Stretcher, etc.
Distance from UMMS facilities	Distances between geo-coded latitudes/longitudes and facility latitudes/longitudes
Employer	Self, County, State, Military, BWMC, Large local employer (Giant Foods, Walmart, BGE, etc.)
Employment Status	Employed/Unemployed this visit, Employed/Unemployed prior visit
English Fluency	Yes/No
ER Frequency	4 Values: 3 Months, 6 Months, 12 Months, 24 Months
Ethnic Group	Standard list
Identifies as Having a Religion	Yes/No
Substance Abuse	Illegal and legal, as a series of binary indicators
Inpatient Days	4 Values: 3 Months, 6 Months, 12 Months, 24 Months
Intubated	Yes/No for this visit, Yes/No for prior visit
Lab Meta	Number of lab orders, number of unique lab tests, etc.
Lab Tests	Min, Max, Mean, and Latest (Na, K, blood counts, hemoglobin, etc.), not limited to this visit
LDA	Lines, drains, and airways (categorized and counted), not including central lines
Legal Substance Abuse	Yes/No current (including Tobacco, Alcohol, etc.), Yes/No historical

© 2019 Morgan DJ et al. *JAMA Network Open.*

Length of Stay	In hours, calculated as date/time of discharge minus date/time of admission
Marital Status	Married, Single, Divorced, Widowed, Separated, Partnered
Medications	Total and categorized (counts by pharmacy class and therapeutic class) this visit and prior visits
Mode of Arrival	Alone/Accompanied, Ambulance, Car, On Foot, Law Enforcement, etc.
Outpatient Visits Attended/Skipped	4 Values: 3 Months, 6 Months, 12 Months, 24 Months
Pain Score	Min, Max, Mean, and Most recent (not limited to this visit)
Post-Discharge Coded Diagnoses	ICD Codes (looking for CKD, COPD, CHF, AKI, Diabetes, Chronic Pain, Mood Disorders, etc.)
Primary Insurance Classification	Self Pay, Medicare, HMO, Military, Commercial, Carefirst/Blue Cross, etc.
Procedure History	Number and types of surgical procedures performed prior to this visit
Procedures	Number and types of surgical procedures performed this visit
Radiology Meta	Number of radiology orders, number of unique radiology study types, etc.
Residence Change	Within the past 2 years represented as two values: Yes/No, and how recent (number of months)
Staff Alerts	VRE, MRSA, VOR, etc.
Mental Health	Categorized "affect", suicide risk, etc.
Ventilator	Yes/No this visit and prior visits along with total vent days
Visit History	Running monthly totals of counts and types of visits within the past 2 years
Vital Signs	Features generated by auto-encoders (HR, Resp, BP, Temp, SaO_2, etc.), not limited to this visit
WDL	Physical Exam "within defined limits" Most recent HEENT WDL, Cardiovascular WDL, Respiratory WDL, etc.

© 2019 Morgan DJ et al. *JAMA Network Open.*
